# Development and process evaluation of a motor activity program for people with profound intellectual and multiple disabilities

**DOI:** 10.1186/s12913-021-06264-z

**Published:** 2021-03-20

**Authors:** Helena J. M. van Alphen, Aly Waninge, Alexander E. M. G. Minnaert, Annette A. J. van der Putten

**Affiliations:** 1 Department of Inclusive and Special Needs Education, University of Groningen, Grote Rozenstraat 38, 9712 TJ Groningen, The Netherlands; 2 Research Group Healthy Ageing, Health Care and Nursing, Hanze University of Applied Sciences, Groningen, The Netherlands; 3 Royal Dutch Visio De Brink, Vries, The Netherlands

**Keywords:** Profound intellectual and multiple disabilities, Motor activity, Implementation, Process evaluation

## Abstract

**Background:**

The support of people with profound intellectual and multiple disabilities (PIMD) rarely focused on motor activity, which might have negative consequences for the quality of life of these people. Evidence-based motor activity programs that present individually tailored and structural motor activity for these people are, regretfully, lacking. This study developed such a program for these people and evaluated the implementation process.

**Methods:**

The motor activity program is developed in accordance with the theoretical premises of the educational program and consists of four methodological steps in which the content is individually filled with: motor activity structurally embedded within the activities of daily living, and 3–5 motor activities aimed at a specific goal, which is evaluated. Program delivery consisted of a manual, explanation to the teams, and coaching of one contact person per participant (*n* = 9). Process evaluation included the delivered fidelity, dose, reach, and adaptations made during the program. In addition, mechanisms of impact and the influence of contextual factors were evaluated. Data collection included researcher logbooks, individual program content, and staff reports.

**Results:**

The intended fidelity, dose, and reach were not obtained in most participants. Content has been made explicit for seven participants, but only in one participant all critical steps in implementation were performed as intended, though later in time. In three participants, previously offered motor activities were described within the weekly program, but without all activities having a clear link with the goal set. It is showed that the core elements of the program were affected with the conceived implementation plan. The time schedule, critical elements in implementation and program content were influenced by a lack of conditions such as professionals’ motivation and responsibility, methodical working, interdisciplinarity and continuity in staff.

**Conclusions:**

The results suggest that the implementation might be improved in case more attention is paid to the organizational conditions and implementation structure. The findings led to substantial changes in the implementation strategy. This study underlines the importance of process evaluation prior to testing for effectiveness.

**Trial registration:**

The (overarching) study was registered at the Netherlands Trial Register (number 6627) on February 10, 2017: https://www.trialregister.nl/trial/6449.

## Background

In order to improve the functioning and participation in daily activities of people with profound intellectual and multiple disabilities (PIMD) [1], stimulating their motor abilities is important. Motor skills play an important role in engagement and interaction of people with PIMD within activities, such as eating and drinking, playing with an object and establishing contact with another person [2–5]. In addition, including motor activities in the support of people with PIMD can be beneficial for both physical (e.g., increasing mobility) and mental health (e.g., decreasing challenging behaviour and increasing alertness) of people with PIMD [3, 6–11].

To date, it seems, however, that structural stimulation of the motor domain is often lacking in the daily support of people with PIMD [4, 12, 13]. Activities offered are usually passive in nature and hardly focus on the motor domain [4, 14–16]. Consequently, the quality of life of these people with PIMD is not targeted by the quality of support. Therefore, more explicit attention should be paid to structural integration of motor activity in the daily support of people with PIMD [4].

The integration of motor activities, however, needs to be well considered in the support of a person with PIMD. The group of people with PIMD is heterogeneous and particularly vulnerable with each person having their own special needs, abilities and preferences [1]. To ensure that not only a structural amount of motor activity, but also the most effective (and not harmful) activities are used for each person with PIMD, individually tailored support, also on the motor domain, is needed [5, 9, 17–21]. In addition, from the view that motor activities can be used to increase the independence and autonomy of these people [4, 5], it is necessary that motor activities are integrated in a methodical and goal-oriented way within an interdisciplinary context to contribute to the individual support objectives of a person with PIMD [12, 20, 22]. As assumed by the educational program [20], which is developed, and has been proven to be effective for people with PIMD, quality support can only be provided to people with PIMD if their capabilities to develop and to build relationships are seen, stimulated and individually approached. Therefore, explicit and planned actions as well as interdisciplinary working are necessary to give full consideration to the needs of these persons [20]. Moreover, to determine the activities that may affect someone’s individual goals, knowledge about the relationships between motor activities and outcomes is needed [3]. Only when there is knowledge of components of motor activities being beneficial on specific outcomes, motor activities can be effectively integrated in the support of people with PIMD.

There are, however, only early indications of the effects of specific motor activities (e.g., aquatics, power-assisted exercises, rebound therapy-based exercises, and psychomotor tasks) for people with PIMD [5, 7, 9, 17–19, 21, 23, 24]. In addition, the effects of motor activities are mostly studied on the motor domain, while motor activities can also be used to achieve outcomes beyond the motor domain, for example on cognitive and social functioning [3, 25]. Therefore, more knowledge is looked-for to tailor the use of motor activities to individual needs and abilities and to integrate them in a goal-oriented way in the daily program of persons with PIMD. In addition, to ensure an optimal quality of support of a person with PIMD, systematic evaluation of outcomes is required [20]. Systematic evaluation of the offered support also increases the knowledge about the relationships between motor activities and specific benefits for persons with PIMD. Unfortunately, it has been shown that most of the activities actually in use for the support of people with PIMD are, regretfully, not systematically evaluated [15, 26].

To date, there is no program that presents motor activity opportunities for structural integration in a methodical, individually focused, and goal-oriented way including systematic monitoring and evaluation of outcomes at an individual level in people with PIMD. Such a program is important to ensure that people with PIMD optimally benefit from motor activity to contribute to the quality of life of these people. In addition, this will increase the available body of knowledge on the relationships between specific motor activities and specific outcomes for people with PIMD. Therefore, the aim of this study was to develop a program which has the purpose to ensure structural and goal-oriented motor activity of people with PIMD within an individual tailored and continuing program and from the perspective that motor activities can contribute also above and beyond the motor domain. In addition, this study aimed to investigate the conditions essential to a successful implementation, by evaluating the process of implementation within the support of people with PIMD at a residential care facility in the Netherlands.

## Methods

### Development of the motor activity program

The motor activity program was designed to ensure that the use of motor activity optimally contribute to, and enhance the quality of, the support of people with PIMD. The ultimate aim of this program is that consideration is given to the way in which motor activity can contribute to the support of the person with PIMD and that motor activity is structurally embedded within the support using a fixed amount of motor activity. The program can be seen as a framework compromising four steps to ensure working in a methodical and goal-oriented way. Within the program, the content should be filled individually with motor activity structurally imbedded within the activities of daily living and 3–5 motor activities aimed at a specific goal. Capturing the content and focus of the activities together with goal-oriented and systematic evaluation, as part of the program, provides measurable insight into the results on an individual level.

#### Theoretical framework

The motor activity program is developed from the view that structural stimulation of the motor domain is a prerequisite for the improvement of the quality of life of persons with PIMD [4]. In addition, the motor activity program is developed in accordance with the theoretical premises of the educational program [20, 27], because of their view on people with PIMD and related way of working to optimally contribute to, and enhance the quality of, support of people with PIMD. The educational program assumes that quality support can only be provided to people with PIMD if their capabilities to develop and to build relationships are seen and individually approached [20]. The program assumes that methodical and goal-oriented working within an interdisciplinary context are required to ensure that the support fits the needs and preferences of each individual with PIMD [20]. The program starts with a profile in which knowledge and views of all those involved in the support are integrated to an individually defined support perspective and related goals formulated in an interdisciplinary context. In addition, within the program it is established in a methodical way how to achieve the goals and what resources are required. Goals are evaluated by making use of a Goal Attainment Scaling [28]. It has been shown that the educational program leads to a better interpretation of the persons’ behavior and a better match between the support and the needs of the person with PIMD. In addition, it has been shown that the educational program increases the collaboration within and between disciplines (and parents) [20].

#### Content of the program

**Fig. 1:**
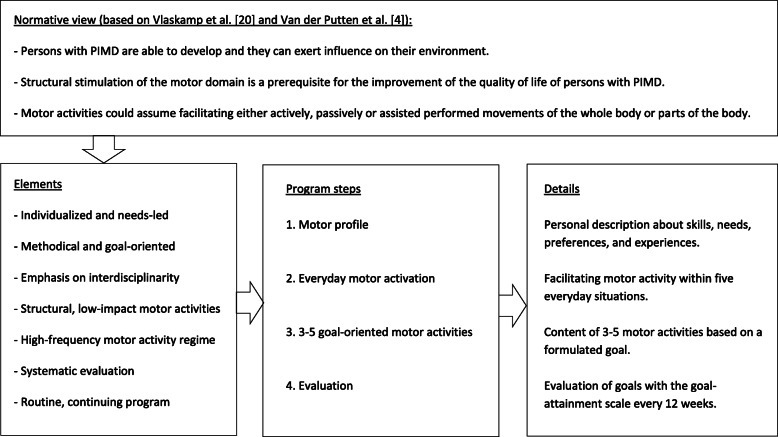
Core elements of the motor activity program including the underlying normative theoretical statements

Based on the theoretical premises of the educational program [20, 27], the motor activity program is processed into four program steps (see Fig. 1) which are designed based on available evidence. *In the first step*, a personal description about individual needs and possibilities on the motor domain is bundled to a motor profile. To create this personal profile, we have relied on the statement of Van der Putten [22] indicating what information should be included within a motor profile. This is processed into a form consisting of seven open questions (including explanation) and a weekly overview of current motor activities. Data source triangulation can be used to collect the required data from different involved professionals and family to gain multiple perspectives and validation of data [29]. *In the second and third step*, based on the motor profile, interdisciplinary consensus is reached on the way of facilitating motor activity of the person with PIMD within five everyday situations (feeding, dressing, grooming and bathing, ambulation, and transfers) [30]. In addition, interdisciplinary consensus is reached on a goal to be formulated and related content of 3–5 motor activities to be integrated within the weekly program [9, 19]. The amount of the motor activity to be integrated in the program is determined based on government and health authorities’ recommendations on the promotion of motor activity and prevention of inactivity [31, 32]. In addition, the number of motor activity opportunities per week has been based on studies showing 3–5 motor activity opportunities per week as feasible and effective on different health and behavioural outcomes in people with PIMD [9, 19]. To determine the specific content within the program, it requires a broad look on motor activity and activities should be individually tuned to meet someone’s needs, preferences, and abilities. In addition, it also requires a broad look on the contribution that motor activity could have as it can be used for a wide range of goals such as goals relating to the motor domain, but also the cognitive and social domain. In order to give direction to the content of motor activities based on individual support objectives, the available evidence base regarding the potential benefits of motor activities in people with PIMD has been used (e.g., [3, 5, 7, 9, 19, 22–24]). As the evidence for the effectiveness of motor activities in the support of people with PIMD appeared to be limited [3, 18], also early indications on content that may be related to specific outcomes have been used to support the choice of activities [18]. This is processed into a form including possible directions for the motor activity (e.g., blood circulation, obstipation, alertness, sleep, challenging behaviour, enjoyment, relaxation, movement experience, self-direction, interaction, balance, muscle strength, functional skills, etc.) together with characteristics for the motor activities that logically contribute to the chosen direction. Moreover, forms are designed to record the goal (including the initial situation and end goal), reporting points and agreements regarding the performance of motor activities. *In the fourth step*, evaluation of the performance of activities and goals took place. After 12 weeks of activity performance, staff reports are evaluated by the use of the goal attainment scale (ranging from no difference compared to the initial situation (0) to accomplishing an end goal (+ 2)) and new goals should be formulated [20, 28].

#### Implementation

The implementation of the motor activity program was designed consisting of three phases including critical elements within these phases and related to a fixed time schedule (see Tables 2 and 3). The three phases are: introduction of the program (12-week period), giving content (8-week period) and actual performance of activities (12-week period). The Quality Implementation Framework of Meyers et al. [33] was used as a reference to include the critical steps for quality implementation within these phases.

##### Introduction of the program

A period of 12 weeks had been assumed to introduce the program to the different stakeholders of the organization (e.g. managers, physicians, healthcare psychologists, and direct support professionals with a coordinating role at the living unit) and to check per participant whether study participation was possible. In addition, the living units and activity groups of the participants had to be informed by the internal physical therapist. Also, the purpose and content of the motor activity program had to be explained during the quarterly team meetings of the living units at which parents or legal representatives were also invited. Next, one contact person (a direct support professional with a coordinating role at each of the involved living units which was familiar with the attending participant) had to be provided with the manual and trained to be able to give content to the program. Therefore, program delivery consisted of a manual (including the resources required for each program step), explanation to the teams, and coaching of one contact person per participant. Within the manual, for each of the program steps it was explained what information is needed, who is responsible (attributed to the location), and how to use the forms. In addition, specific attention within program delivery was paid to the normative view and program steps to ensure methodological and interdisciplinary working and monitoring and evaluation of implementation and goals.

##### Giving content

A period of 8 weeks had been assumed to give content to the individual programs before actual performance of, and reporting on, the activities (see Table 2). The contact person agreed to collect and bundle the required information from involved professionals and family for the motor profile and to schedule an interdisciplinary meeting (i.e. a meeting with at least a representative of the living unit and activity group, the physical therapist, and health care psychologist). To save time with regard to the motor profile, already archived information within individual case files was collected and bundled by the first author prior to the collection of data from the involved professionals. The health care psychologist was responsible for the final content of the motor profile and for goal setting based on the perspectives of all professionals involved. Direct support professionals in collaboration with the physical therapist (or movement teacher) were responsible for the content of motor activities.

##### Actual performance of activities

A period of 12 weeks had been assumed to perform the motor activities and to report on the goals and activities. Performance of, and reporting on goals and activities, were the responsibility of direct support professionals at the living unit and activity group and the physical therapist and/or movement teacher. After 12 weeks, evaluation is based on the Goal Attainment Scale and new goals should be formulated within an interdisciplinary context. Because it was not allowed to make adaptations to the online reporting system during pilot testing, description and reporting on activities and goals had been assumed with the forms of the motor activity program printed out and bundled in a folder.

### Pilot testing and modelling process and outcomes

#### Design and participants

To evaluate the implementation of the motor activity program, a pilot study with a descriptive multiple-case design [34] was conducted at a large-scale 24-h residential care facility in the Netherlands. Nine participants with PIMD participated. Participants were recruited based on eligibility within the facility and recruited by a physical therapist based on the criteria from Nakken and Vlaskamp [1]: profound intellectual disability (intelligence quotient (IQ) under 25 points or a developmental age of up to 24 months) and severe or profound motor disability (classified as Gross Motor Function Classification System (GMFCS) IV or V [35]). Table 1 shows the age, gender, mobility, health problems and context of the participants. Participants with suspected dementia and a living situation outside the facility were excluded. The parents or legal representatives of the participants provided written informed consent and the study was approved by the ethical committee for Pedagogical and Educational Sciences of the University of Groningen, the Netherlands. Moreover, the study was exempted for medical approval by the Medical Ethical Committee of the University Medical Center Groningen, the Netherlands.

**Table 1:** Age range, gender, mobility, health problems and context of the participants

Participant	Age range (years)	Sex	Mobility	Health problems	Living unit ^b^	Activity group
1	17–30	Female	Fully wheelchair dependent	Blindness, epilepsy	1	n.a. ^a^
2	17–30	Female	Fully wheelchair dependent	Blindness and auditory impairment, epilepsy	1	n.a. ^a^
3	17–30	Female	Fully wheelchair dependent	Blindness and auditory impairment, epilepsy	2	1
4	Above 45	Female	Fully wheelchair dependent	Visual and auditory impairment	3	2
5	17–30	Male	Fully wheelchair dependent	Visual impairment, epilepsy	3	2
6	31–45	Male	Fully wheelchair dependent	Blindness and auditory impairment, epilepsy	4	3
7	Above 45	Female	Requires heavy assistance to mobilize	Visual and auditory impairment	5	4
8	17–30	Female	Fully wheelchair dependent	Blindness, epilepsy	6	2
9	17–30	Female	Fully wheelchair dependent	Visual impairment, epilepsy	6	5

^a^ Note. Participant 1 and 2 received activities at their living unit

^b^ The same contact person was involved of participants from the same living unit. Also of participant 3 and 6, the same contact person was involved

### Data collection

The process evaluation was guided by the framework of the Medical Research Council [36] and focused on three key components: implementation, mechanisms of impact, and context.

*Implementation* was studied with regard to the time schedule, critical elements in implementation, and content of the program in terms of implementation fidelity (whether the program was implemented as intended), implementation dose (the quantity of the program implemented), reach (involvement of devised responsible staff), and adaptations made [36]. *Mechanisms of impact* referred to the participant outcomes based on the program content and performance and understanding of unexpected pathways and consequences. *Context* referred to the factors that may have affected the implementation and outcomes. Qualitative and quantitative data were gathered during implementation using the following data sources: individual program content of the motor activity program (number and content of activities, goal description, and reporting points), staff reports and evaluations as part of the program (number and content of reports on the goal and performance of activities, including the reasons when not performed, and the Goal-Attainment Score), and a logbook which included for each of the participants the role of the researcher with all dates of contact with the contact person and other involved professionals, the time schedule of the steps taken, and observed barriers directed by selected potential factors (professionals’ view and motivation, methodical working, interdisciplinarity, tasks and responsibility and continuity in staff) [20, 37].

#### Data analyses

Descriptive statistics (frequencies and percentages) were applied to the quantitative data (number of activities integrated within the weekly program, number of reports, number of activity performance, and percentage of motor activities that have been performed as planned). Documentary and content analysis were performed on the logbook, individual program content, and staff reports. This documentary and content analysis focused on the execution of the program compared to the intention of the program with regard to the time schedule, critical elements in implementation and content of the program. It was analysed whether the critical elements in implementation and the content of the program were executed as intended (+), executed but not entirely as intended (+/−), or not executed (−). It also focused on intended outcomes vs. actual outcomes and reasons if motor activities were not performed. We also evaluated whether the devised responsible staff were involved in the implementation phases and related critical elements of implementation. In addition, the factors that may have affected the implementation and outcomes were described.

## Results

### Implementation

#### Fidelity, dose, reach, and adaptations made

During *introduction of the program*, all involved stakeholders were informed and agreement was given for participation. In addition, the quarterly team meetings of the living units were attended to further explain the program. In one case, however, the quarterly team meeting occurred later than intended, because of unforeseen circumstances (i.e. a funeral) (see Table 2). In addition, in the case of two participants, an extra team meeting was organized to present the program, but only two team members instead of the whole team turned out to be present (see Table 3, participant 8 and 9). Moreover, at the team meeting of two participants (participant 4 and 5), there was clearly some resistance with regard to the time investment of the program and overlap with previous projects. Despite, for each of the participants a contact person as intended was reached and coached for about 3–4 h in total on the different steps of the program. A total of five contact persons were involved, of which four were related to two participants each, and one contact person with one participant (see Table 1).

**Table 2:**
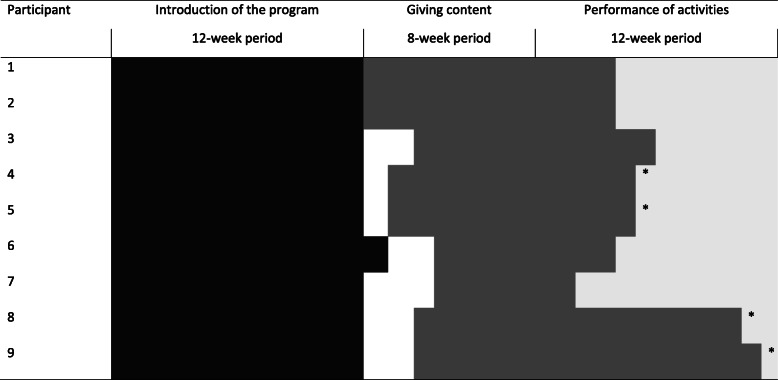
The implementation fidelity with regard to intended time scheduled and the time period used for introduction of the program, giving content, and performance of activities

The black filling shows the time period used for introduction of the program

The grey filling shows the time period used for giving content

The (semi) white filling shows the time period used for the performance of activities

* Performance of, and reporting on, motor activities has taken place for these participants while giving content was incomplete at that moment

**Table 3:** The implementation fidelity with regard to the implementation steps and content of the program

Participant	Introduction of the program				Giving content			Performance of activities
	Organization wide	Activity group	Living unit	Training of contact person	Information bundled to the motor profile	Interdisciplinary meeting	Description of activities and goals	Performance of, and reporting on, activities
1	+	n.a.^a^	+	+	+	+/−	+/−	+ (?)^a^
2	+	n.a.^a^	+	+	+	+/−	+/−	+ (?)^a^
3	+	+	+	+	+	+/−	+	+
4	+	+	+	+	+/−	–	+/−	+/−
5	+	+	+	+	+/−	+/−	+/−	+/−
6	+	+	+	+	+/−	+/−	+	+
7	+	+	+	+	+	+	+	+
8	+	+	+/−	+	+/−	–	–	+/−
9	+	+	+/−	+	+/−	+/−	–	+/−

Note. + means executed as intended, +/− means executed but not entirely as intended, − means not executed

^a^ Participant 1 and 2 received activities at their living unit. In addition, for these participants reporting forms of the motor activity program were lost at the living unit after 8 weeks of activity performance

All contact persons agreed to collect and bundle the required information for the motor profile and to schedule an interdisciplinary meeting. Two contact persons related to four participants (participant 4 and 5 and 8 and 9), however, were unable to fulfill the information for the motor profile and to organize the interdisciplinary meeting. One had to delegate the tasks because she changed jobs and the other contact person conveyed the tasks unclearly (based on communication with involved staff) and was unreachable for a certain period. For these four participants, in consultation with management, assistance by one of the researchers (first author) was given to continue the implementation process by organizing and joining a meeting with a participant representative of the living unit and a participant representative of the activity group. For two of these participants, it was forced to have a meeting with the representative of the living unit and activity group separately, because of non-matching working hours. They were in contact by e-mail and in one case they subsequently involved the health care psychologist to give content to the program. As shown in Tables 2 and 3, however, none of the intended critical steps in implementation of the phases “giving content” and “performance of activities” were entirely executed as intended for abovementioned four participants (participant 4, 5, 8 and 9).

For most other participants, setting up the motor profile and interdisciplinarity were not entirely executed as intended. Only in the case of four participants (44.4%) the health care psychologist was involved in setting up the motor profile (see Table 3). In addition, in the case of three participants (participant 3, 6 and 7) an interdisciplinary meeting has been scheduled by the contact person, but only in one case the meeting has actually taken place within the interdisciplinary context as intended (see Table 3, participant 7). For the other two participants, the program content and goals were informed by e-mail to those who could not attend the meeting (in this case the physical therapist and healthcare psychologist). In the other two cases (participant 1 and 2), the program content and goals were solely discussed by the contact person and involved professionals by e-mail.

For all participants (100.0%), it took longer than intended to *give content* to the programs (see Table 2). Content has been made explicit in the programs (i.e. goals and motor activities) of seven participants (77.8%) within the period of pilot testing (see Table 3). For these seven participants, content has been made explicit for all intended everyday situations and the 3–5 motor activities integrated within the weekly program. With regard to the formulation of goals, in one case it was neither described with reference to the initial situation (0) nor to an end goal (+ 2) as intended.

Because of the delayed time schedule for giving content, *performance of the program* started later in time and for none of the participants the program has been fully performed within the timetable set for pilot testing (see Table 2). In the case of five participants (55.6%) the performance and reporting on activities has started as intended although a few weeks later (see Tables 2 and 3). For these participants, six to 10 weeks have been reported on the performance and outcomes of the motor activities. In the case of two participants (participant 1 and 2), however, the reporting forms of the motor activity program were lost at the living unit after 8 weeks of activity performance (probably due to renovation at the unit at that time). Based on the logbook of the researcher it can only be suggested that direct support professionals reported on the performance and outcomes of the activities of these participants. Based on the number of motor activities integrated within the weekly program of the other three participants (participant 3, 6, and 7), 41, 48, and 93 reports were expected instead of the 21, 20, and 76 reports actually available. Based on the reports, the motor activities have been performed as planned for 46.1, 80.0, and 90.5% of the time.

For the other four participants (44.4%) the performance and reporting on activities went different from intended (see Table 3). The original conceived reporting forms were not used and there was no interdisciplinarity among different professionals as the activity group and living group did not start with reporting at the same time (see Tables 2 and 3). Moreover, although for two participants the goals and motor activities were not described, some performance of, and reporting on motor activities has taken place at the activity group (see Tables 2 and 3, participant 8 and 9). As none of the participants completed the 12 weeks of activity performance, Goal Attainment Scores were missing and evaluations within an interdisciplinary context did not take place within the study period.

### Mechanisms of impact

Table 3 shows that essential steps in implementation are not well understood. Only in the case of participant 7 all implementation steps were entirely executed as intended, though later in time. In addition, the number of performed motor activities lagged behind the number of planned motor activities, which influences the outcomes on the level of motor activity and related health and behavioural outcomes. It also influences the possibility of evaluation on an individual level. However, as the programs of the participants, of which we were able to evaluate the staff reports, were focused on gathering knowledge of specific motor activities on specific outcomes, first insights could be obtained for these participants. Despite the low number of available staff reports, it can be suggested that the alertness of the relevant participant improves with an increased active involvement and change in body posture integrated within the motor activities. In addition, it can be suggested that in one case, one specific motor activity clearly stands out when it comes to pleasure for this particular participant. In another case, it can be suggested that independency during the motor activities for this participant relates to his alertness and muscle tension.

Based on the content of the filled programs also some unexpected pathways were investigated affecting the outcomes of the program. The everyday situations, in some cases, were not described in relation to facilitating motor activity. For instance, the feeding situation was not described in relation to motor activity with the argument of tube feeding. In addition, in the case of four participants, ambulation (i.e. relocation) was described in relation to involvement in general, and not facilitating motor behaviour, with the argument of the participant being unable to contribute motorically. In addition, in the case of two participants, the way of facilitating motor activity within the 3–5 motor activities integrated in the weekly program included one of the everyday situations already described within the program. In addition, the duration and moment of performance of the motor activities were not described for two of the attending participants. With regard to the content in relation to the goals set, in three participants, previously offered motor activities were described, but without all activities having a clear link with the goal set. In addition, the previously offered motor activity within the weekly programs of four participants (44.4%) were substantially changed after implementation of the program. In the other cases, no changes were made although the already offered 3–5 motor activities were now more explicitly focused because of the goal set.

### Context

The context likely influenced the implementation and outcomes. It is showed that “contextual problems” in the first steps (e.g., discontinuity in staff, resistance to the program, and a team meeting with only two members being present) directly leads to a program without collaboration between professionals and performing of next steps while previous steps are incomplete (see Tables 2 and 3). In addition, non-matching working hours between staff from the activity group and living unit hampered interdisciplinary collaboration. Moreover, the contact person had a very decisive role. For the motor profile, the collection and bundling of the required information appeared to be dependent on the contact persons’ motivation and responsibility to communicate the importance and required information, and to gain multiple perspectives and validation of data. For example, in the communication about the motor profile, for six out of nine participants (66.7%), the emphasis was on the research purpose, while the interest of supporting the participant was not made clear. In addition, professionals’ motivation and responsibility to perform the program also emerges within the staff reports containing the reasons for not performing the motor activities. Reasons were for instance a different activity program at the time of the planned motor activity, problems with the material (material at a different location or does not work), forgetting to perform the activity, or another activity at the same time.

## Discussion

### Findings

The aim of this study was to develop a motor activity program to ensure structural and goal-oriented motor activity of people with PIMD and to examine the implementation process at a residential care facility. This study described the components of the program and investigated the conditions essential to a successful implementation. Program development has resulted in an individually tailored motor activity program with core components such as individualized and needs-led, methodical and goal-oriented, emphasis on interdisciplinarity, and structural motor activity. With the current implementation strategy, however, the intended fidelity, dose, and reach were not obtained in most participants. Content has been made explicit for seven participants (77.8%), but only in one participant (11.1%) all critical steps in implementation were performed as intended, though later in time. In addition, only of three participants (33.3%), staff reports on the performance and outcomes of motor activities could be evaluated. It is showed that the core components of the program were affected with the conceived implementation plan. The time schedule, critical steps in implementation and program content were influenced by a lack of conditions such as professionals’ motivation and responsibility, methodical working, interdisciplinarity and continuity in staff, leading to a decreased implementation fidelity and dose.

### Theoretical reflection and implications

The core components of the motor activity program are based on normative and theoretical statements which cannot be dissociated from the program. Methodical and goal-oriented working with explicit and planned actions as well as systematic evaluation within an interdisciplinary context are required to ensure that the support fits the needs and preferences of each individual with PIMD [27]. Based on the process evaluation it appears, however, that the core components of the program were at risk with the current way of implementation, in particular at the phase of giving content. Results showed that the motor profiles of five participants may have been incomplete or invalid by not involving the perspectives and findings from all different disciplines [29]. In addition, for eight participants, a lack of interdisciplinarity emerged at the planned meeting while intended to serve as the starting point for giving content to the programs [20]. This may have led to programs based on someone’s opinion or intuition instead of a well-grounded support plan [22]. In addition, this lack of interdisciplinarity may have created fragmentation and discontinuity of the support which puts the relationship with the person under pressure [20]. This is concerning as it could keep the person with PIMD from the most effective or pleasurable motor activities targeting the quality of life of people with PIMD. Also the lack of, or incomplete description of activities has impact on the quality of the support. This incompleteness makes it unclear what actually has been performed and where the outcomes are actually based upon [15, 18]. Moreover, without interdisciplinarity among different professionals and without a description of activities and goals, the performance of, and reporting on activities does not contribute to evaluation aimed at promoting the quality of life of persons with PIMD. Therefore, evaluation of the effectiveness of the program on behavioural and health related changes would have been meaningless, because the implementation fidelity and dose could already explain the lack of effectiveness making the effect of the Program Concept And Underlying Theory Unclear [38]. It is showed in the literature that there is a lack of evidence-based motor interventions that have been proven to be effective for specific domains of people with PIMD [3, 18, 22]. An explanation may be that there is little evidence on the effectiveness of motor activities due to problems in the process of implementation in practice. The current study showed some insights with regard to the mechanisms of impact of the program and specific motor activities and outcomes, because of goal-oriented working and monitoring of activities and goals. This confirms that evaluation of motor activity support on an individual level will increase the available body of knowledge on the relationships between specific motor activities and specific outcomes for people with PIMD. Future studies can focus on hypothesis testing with regard to the amount of motor activity and the specific content of motor activity and its outcomes. Dependent on the initial level of motor activity integrated within the support it is expected that, with the motor activity program, the amount of motor activity of people with PIMD will increase and/or that the content of motor activities will be adapted to better suit the needs of the person with PIMD. In the current study, as a result of goal-oriented working, the previously offered motor activity within the weekly programs of at least four participants were substantially changed after implementation of the program. Based on the content of motor activities, specific outcomes can be logically reasoned and checked based on staff reports and measurable results from instruments. In future studies, instruments such as diaries and accelerometers can be used to evaluate the motor activity of each individual with PIMD before and after implementation [39]. Moreover, instruments related to the goals set can be used to prove the findings based on staff reports.

To improve the implementation process of the motor activity program in practice, it is important that more attention is paid to the structure for implementation [33]. As the current way of implementation largely relied on one direct support professional (the contact person), a training program for a team of professionals could improve the implementation of the motor activity program [33, 40]. Implementation teams make the implementation process less vulnerable for one professionals’ individual view, capabilities and responsibility as well as for discontinuity, such as holidays and the move into other jobs [37, 40]. In addition, implementation teams have a shared responsibility for the implementation of the program with regard to processes and timelines [33]. Moreover, it is shown that the capability and motivation levels of direct support professionals could be improved with additional training in motor activity support [40]. Bossink and colleagues [40] have showed, for instance, that direct support professionals find it hard to structurally integrate motor activity within the daily program of people with PIMD, because they lack the required knowledge and skills. Not meeting the intended time schedule in the current study may be related to the capability of direct support professionals. Worksheets with exercises to practice and spread over training days, such as designed in the study of Hanzen [41], may improve the implementation of the motor activity program.

To ensure that the motor activity program will be implemented with quality, the core components of the program should be leading in a training program for a team of professionals. Attention should be paid to the organizational conditions to enable the organization to function better in activities such as an interdisciplinary collaboration and in procedures to achieve the goals (e.g., methodical working, reporting and evaluating) [33]. In addition, to overcome that the performance of motor activities remained unreported or will be replaced by different activities, training should also be sufficient in teaching the “why” regarding the specific tasks of the motor activity program [33]. Moreover, the view on motor activity for people with PIMD needs consensus among professionals to fully ensure structural integration of motor activity and overcome involvement in general without active or passive movement participation [18].

#### Strengths and limitations

The main strength of this study is that an innovative theory-based motor activity program designed to improve the structural and goal-oriented motor activation imbedded in the support of people with PIMD, was implemented in practice. In addition, the process evaluation enabled the identification of factors crucial to preserve the core components of the intervention. In addition, the results provides suggestions to improve future implementation. Moreover, the current results also showed the added value of goal-oriented and methodological working and evaluation of motor activity at an individual level. As there was no program that presents motor activity opportunities for structural integration in a methodical, individually focused, and goal-oriented way including systematic monitoring and evaluation of outcomes at an individual level in people with PIMD, the results form an important step toward evidence based support for motor activity for people with PIMD.

The main limitation of this study is that it represents the implementation process in the context of one residential facility, so the results were strongly influenced by the initial situation with regard to the current way of working and based on the vision on the target group as well as on the importance of motor activation. As several projects aimed at motor activity and lifestyle had already been carried at this facility it could be that the added value of capturing the activities and goal-oriented and systematic evaluation should have been more emphasized in order to see its value. In addition, if the facility had already worked with the educational program, this would most likely have facilitated the implementation fidelity and dose. It is not clear whether implementation at another residential facility would have led to similar results. In addition, the implementation process of the motor activity program possibly also improves if all persons from one living unit or activity group are involved within such innovations. The nine participants with PIMD that participated in the current study were recruited based on the criteria from Nakken and Vlaskamp [1]. Due to the study inclusion criteria, the number of persons participating per living unit and activity group varied from one to three participants (see Table 1), while all groups contained about eight persons with a variety of capabilities. Although the conceived program is individually tailored, it would probably have helped to involve whole groups in the implementation of such new innovations. We can learn from the results and considering the crucial factors in future implementation, but the results cannot be generalized.

Moreover, although we would argue that the researchers were close enough to the implementation of the program to record crucial problems and to track why these occurred after all [36], this study only included data facts from a researchers perspective. This limited the data collection and information on the experiences of staff are, regretfully, lacking in the current study. For future studies we recommend evaluation questionnaires to expand the process evaluation. In addition, we can learn from studies and their data sources that describe process evaluations in a similar context [41].

Another limitation is that the logbook data of the current study were originally intended to be better able to interpret the outcomes of the program during pilot testing. Specific factors have been noted, but data are limited to what has come to the attention of the researcher. Although the first author was on location almost every day during implementation, the logbook data are limited to those that came across at the work floor and which were came up during the contacts with the staff involved. Different methods of data collection and a more thorough devised implementation plan with barriers seen by the location beforehand would probably have led to a more specific data collection. For future research, it is recommended that the process evaluation includes broader data from all stakeholders involved in the development and implementation of the program [36]. This may lead to other conditions essential to successful implementation or another understanding why problems occurred during implementation of such a motor activity program [36].

Another point to bear in mind is that although the conceived program has been based on a social-behavioural framework [20], the process evaluation of the current study was guided by the framework of the Medical Research Council. This framework aimed to provide guidance on process evaluation of complex public health interventions [36, 42], such as promoting physical activity. We considered the Medical Research Council framework as highly relevant, because it is recently updated, widely cited and intended to help researchers to think themselves what aspects of the program and context should be taken into account [36, 42]. Also social frameworks such as the implementation impact assessment of Chen [43] could have been taken into consideration for process evaluation. However, based on the assumption that process evaluations will vary, but that they compromise comparable considerations during program development and planning of the evaluation [36], we suggest that different frameworks would have featured similar main findings. However, it may be a limitation with regard to testing the underlying theory because of the different theoretical perspective and framework.

Another point to bear in mind is that the forms of the motor activity program had to be used on paper, because it was not allowed to make adaptations to the online reporting system during pilot testing. Although conclusions cannot be drawn from current data, we suggest that the lack of reporting and performance of activities may have been hampered by inability to use the standard online reporting system. To improve future applications, it is important that we learn from experience and that such resources as the standard reporting system should be available and adapted to innovations [33].

## Conclusions

This study presented the development and evaluation of a motor activity program including the lessons learned during implementation. The findings led to substantial changes in the implementation strategy with the recommendation of a training program with specific content for a team of professionals. Moreover, it also indicates that motor activity for people with PIMD can contribute also beyond the motor domain, for example on pleasure, independence and alertness. Further studies can build on these findings towards quality implementation and to increase the knowledge about the relationships between motor activities and specific benefits for persons with PIMD.

## Data Availability

The data analysed during the current study are not publicly available, but are available from the authors on reasonable request.

## Abbreviation

PIMD: Profound intellectual and multiple disabilities
